# Supplementary material for: Formation of chlorinated breakdown products during degradation of sunscreen agent, 2-ethylhexyl-4-methoxycinnamate in the presence of sodium hypochlorite
Source: Environ Sci Pollut Res Int. 2015 Sep 26;23:1886–97. doi: 10.1007/s11356-015-5444-0 (PMC4713459; doi:10.1007/s11356-015-5444-0)
Supplement: Supplementary file 1 — (PDF 238 kb) [file 11356_2015_5444_MOESM1_ESM.pdf]

<u>**Supplementary Materials**</u>

Formation of chlorinated breakdown products during degradation of sunscreen agent, 2-ethylhexyl-4-methoxycinnamate in the presence of sodium hypochlorite

Alicja Gackowska[a], Maciej Przybyłek[b*], Waldemar Studziński[a] and Jerzy Gaca[a]

[a] *Faculty of Chemical Technology and Engineering, University of Technology and Life Science, Seminaryjna 3, 85-326 Bydgoszcz, Poland*

[b]*Department of Physical Chemistry, Collegium Medicum, Nicolaus Copernicus University, Kurpińskiego 5, 85-950 Bydgoszcz, Poland*

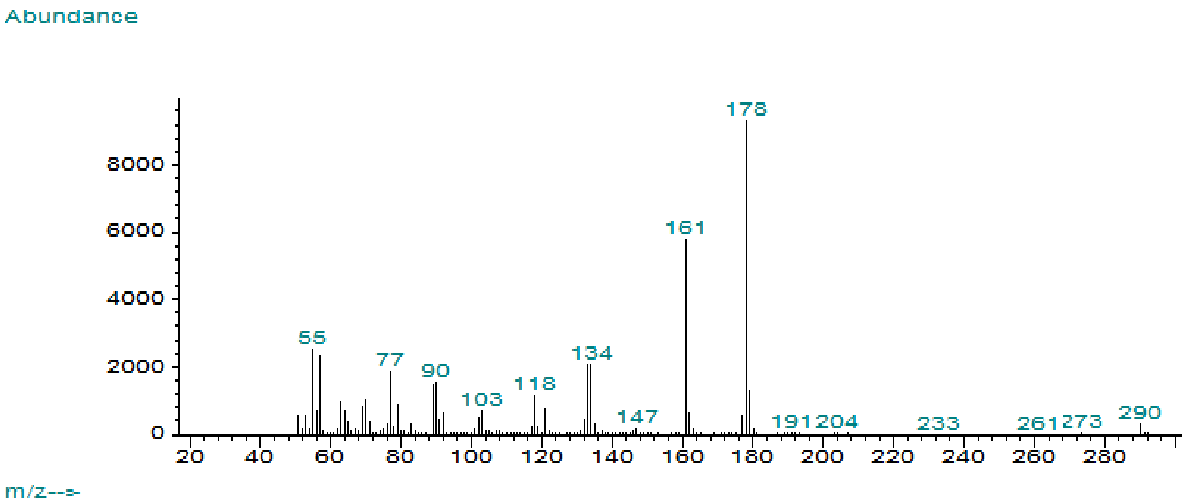

Fig.S1 Mass spectra of Z-EHMC detected in the EHMC/NaOCl reaction mixture

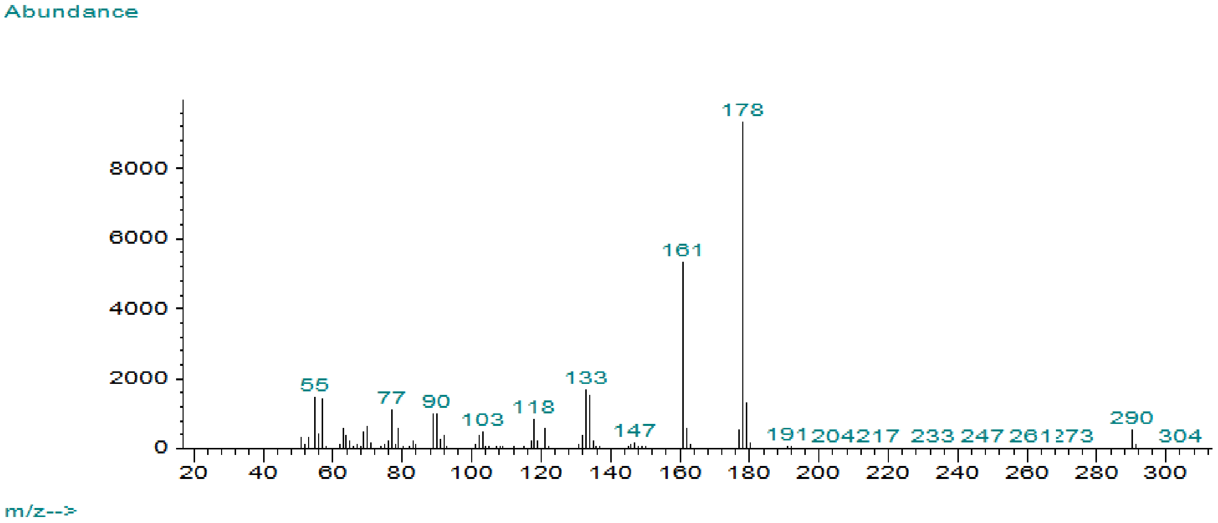

Fig.S2 Mass spectra of E-EHMC detected in the EHMC/NaOCl reaction mixture

* Corresponding author. m.przybylek@cm.umk.pl, tel. (52) 585-36-78

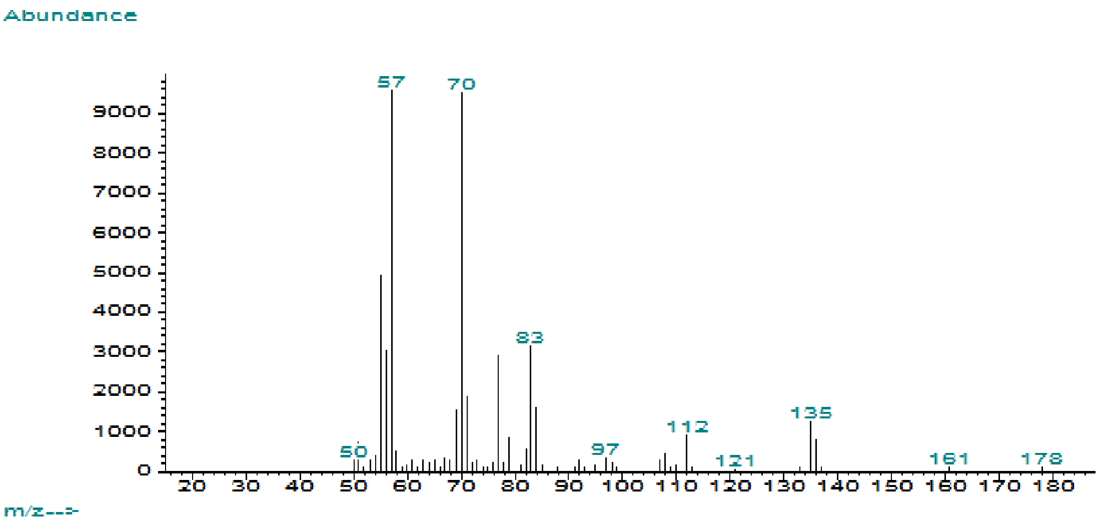

Fig.S3 Mass spectra of 2-ethylhexyl chloroacetate detected in the EHMC/NaOCl reaction mixture

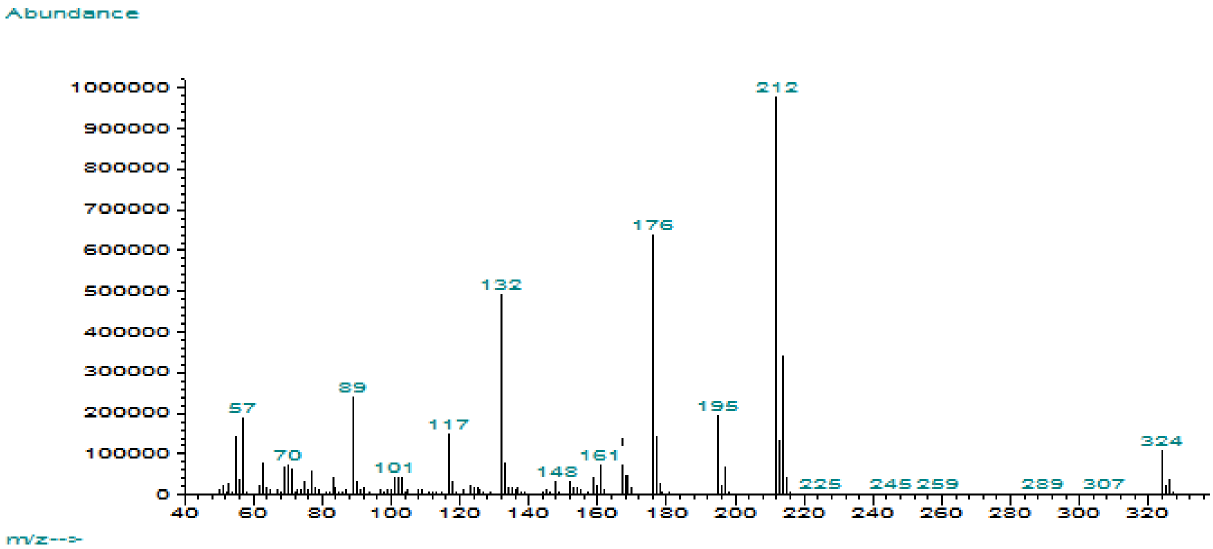

Fig.S4 Mass spectra of 1EHMCCl detected in the EHMC/NaOCl reaction mixture

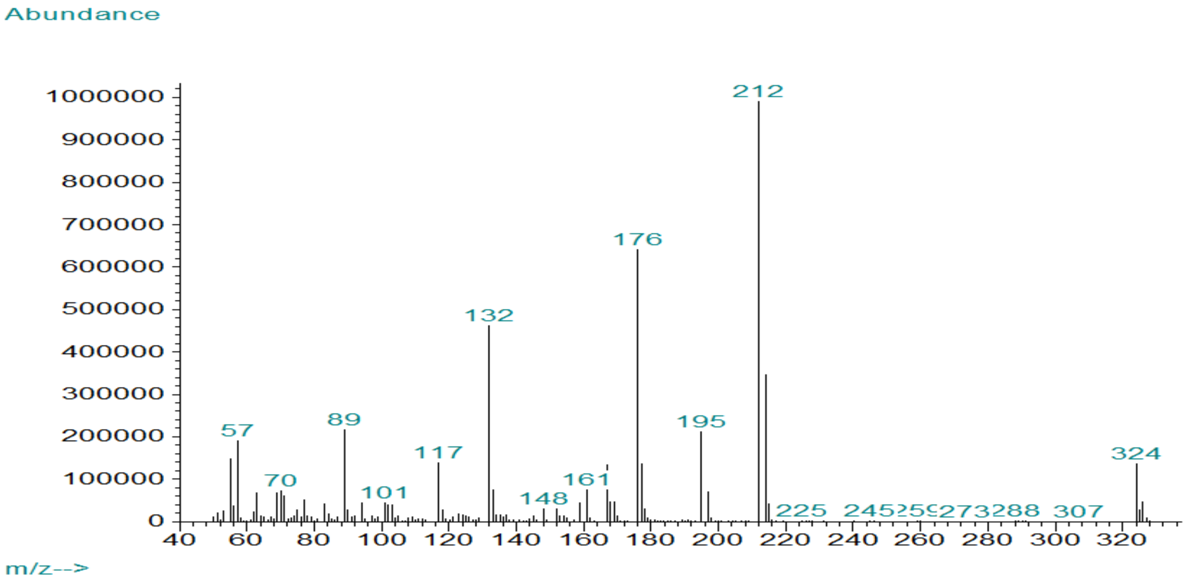

Fig.S5 Mass spectra of 2EHMCCl detected in the EHMC/NaOCl reaction mixture

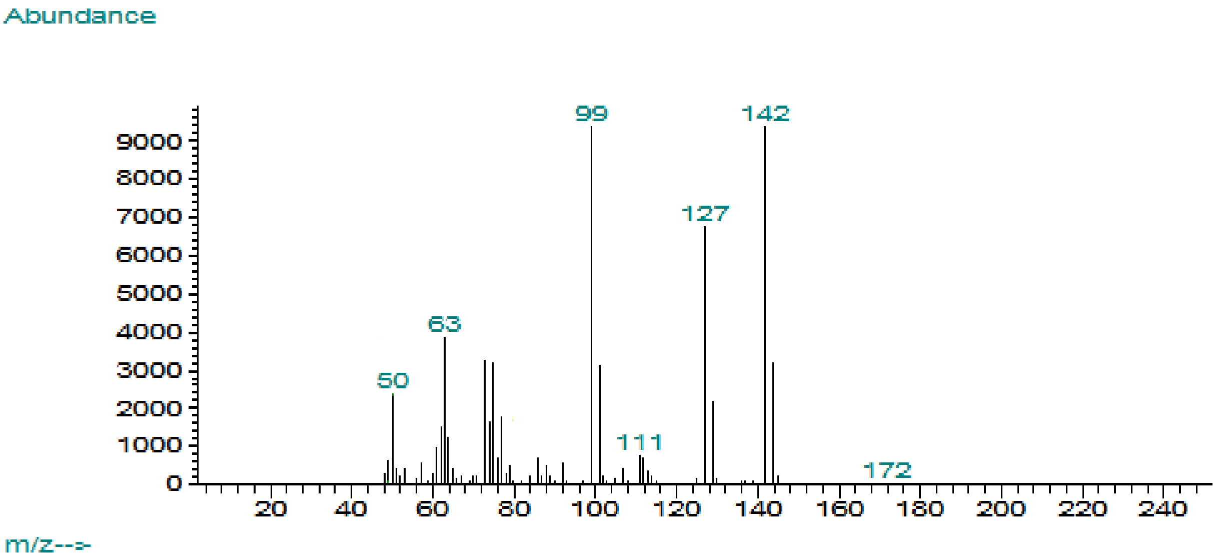

Fig.S6 Mass spectra of 1-chloro-4-methoxybenzene detected in the EHMC/NaOCl reaction mixture

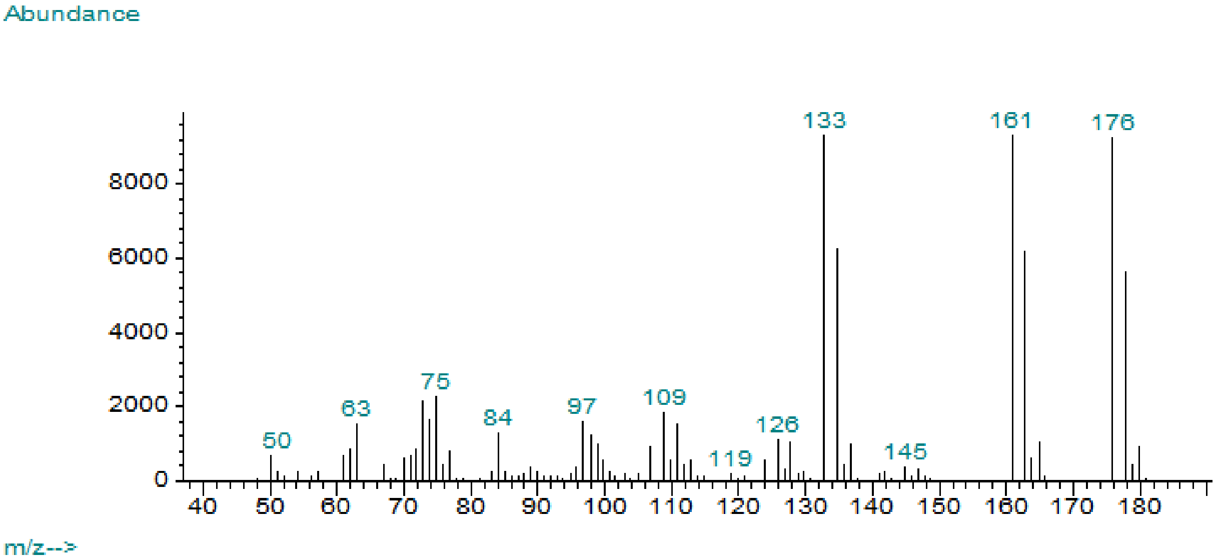

Fig.S7 Mass spectra of 1,3-dichloro-2-methoxybenzene detected in the EHMC/NaOCl reaction mixture

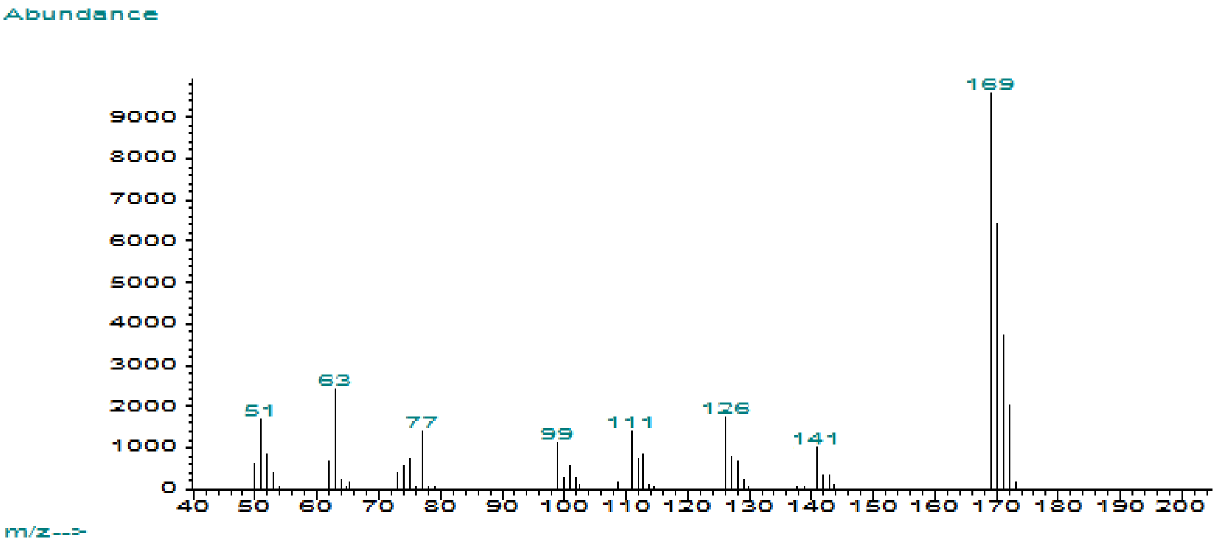

Fig.S8 Mass spectra of 3-chloro-4-mehoxybenzaldehyde detected in the EHMC/NaOCl reaction mixture

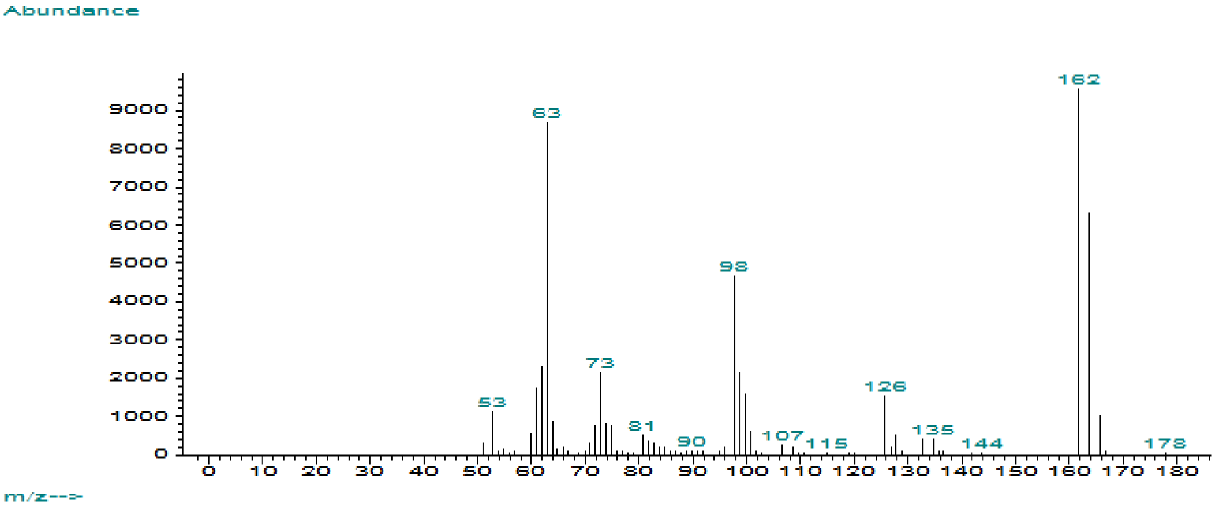

Fig.S9 Mass spectra of 2,4-dichlorophenole detected in the MCA/NaOCl reaction mixture

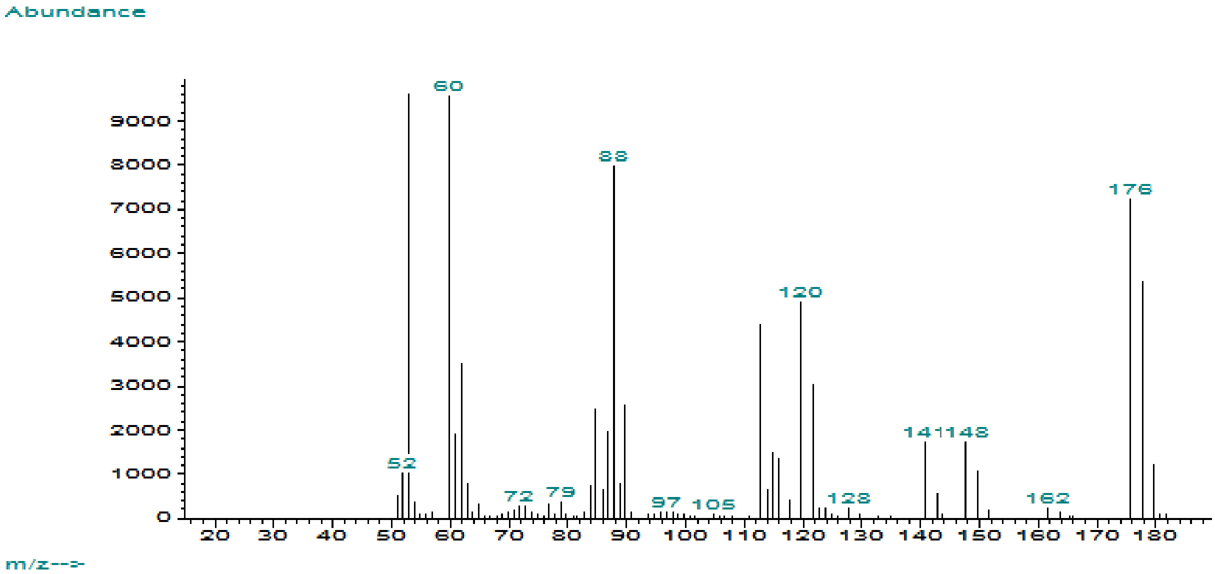

Fig.S10 Mass spectra of 2,6-dichloro-1,4-benzoquinone detected in the MCA/NaOCl reaction mixture

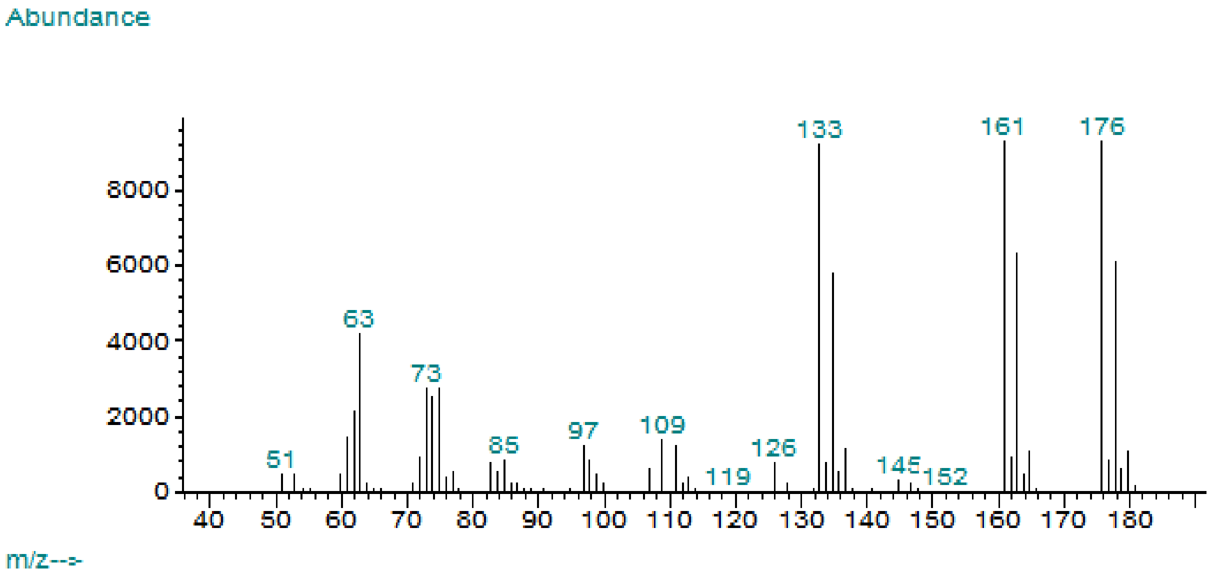

Fig.S11 Mass spectra of 1,3-dichloro-2-methoxybenzene detected in the MCA/NaOCl reaction mixture

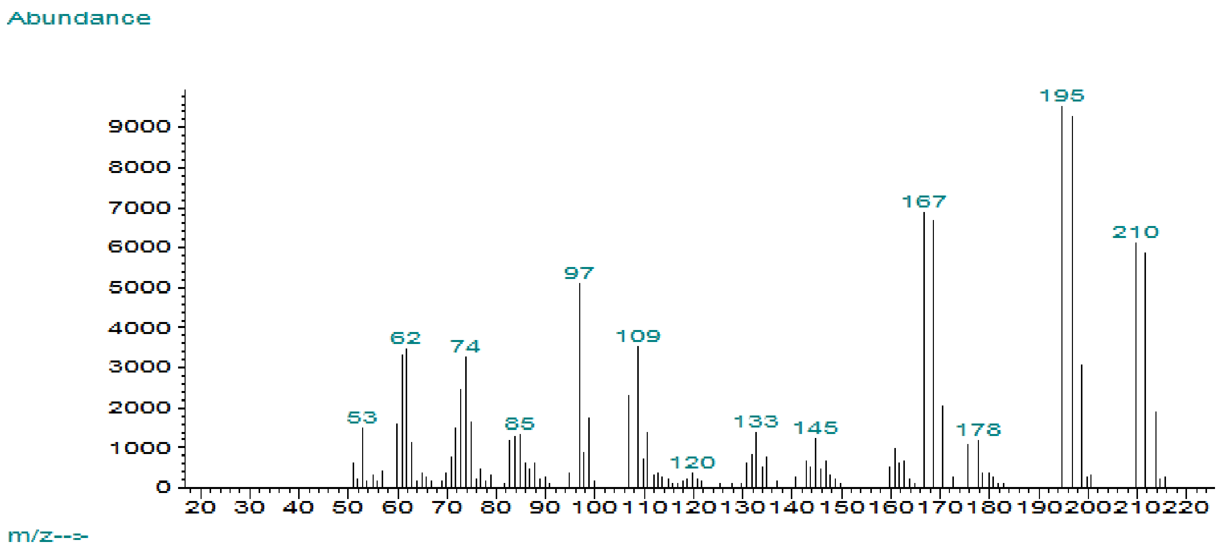

Fig.S12 Mass spectra of 1,2,4-trichloro-3-methoxybenzene detected in the MCA/NaOCl reaction mixture

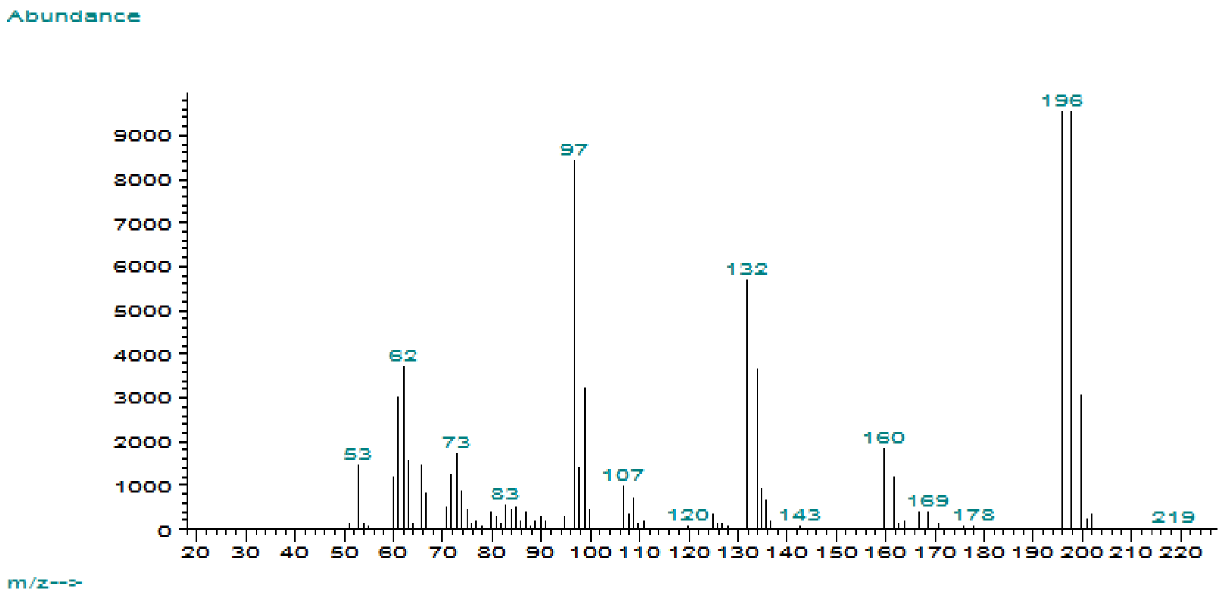

Fig.S13 Mass spectra of 2,4,6-trichlorophenole detected in the MCA/NaOCl reaction mixture

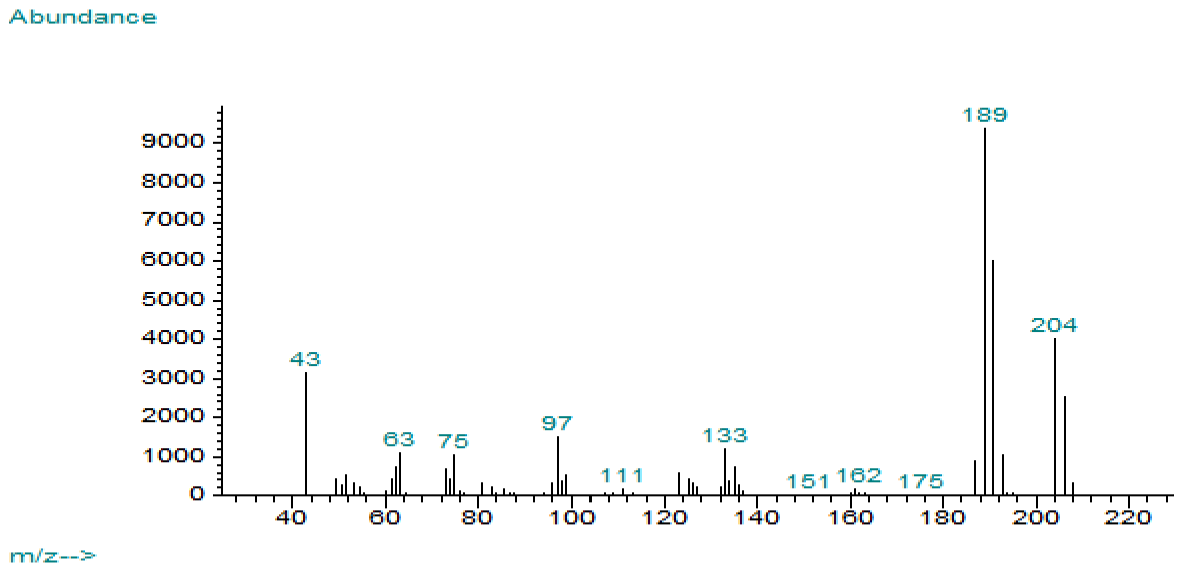

Fig.S14 Mass spectra of 3,5-dichloro-2-hydroxyacetophenone detected in the MCA/NaOCl reaction mixture

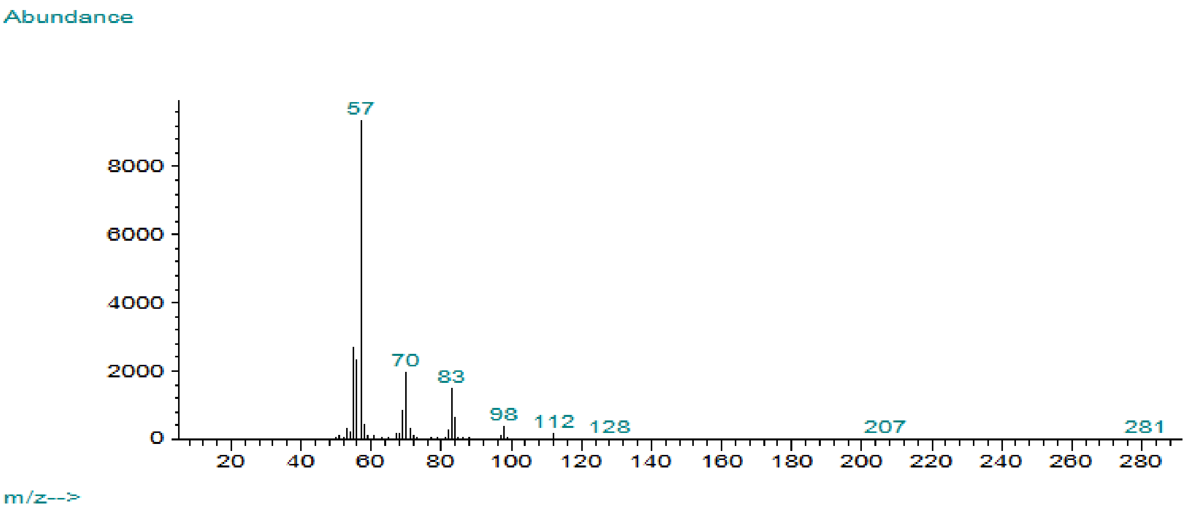

Fig.S15 Mass spectra of 2-ethylhexyl alcohol detected in the EHMC/UV reaction mixture
